# Regulation of cell survival by sphingosine-1-phosphate receptor S1P_1_ via reciprocal ERK-dependent suppression of Bim and PI-3-kinase/protein kinase C-mediated upregulation of Mcl-1

**DOI:** 10.1038/cddis.2013.455

**Published:** 2013-11-21

**Authors:** C Rutherford, S Childs, J Ohotski, L McGlynn, M Riddick, S MacFarlane, D Tasker, S Pyne, N J Pyne, J Edwards, T M Palmer

**Affiliations:** 1 Institute of Cardiovascular and Medical Sciences, College of Medical, Veterinary and Life Sciences, University of Glasgow, Scotland, UK; 2 Strathclyde Institute of Pharmacy and Biomedical Sciences, University of Strathclyde, Glasgow, Scotland, UK; 3 Institute of Cancer Sciences, College of Medical, Veterinary and Life Sciences, University of Glasgow, Scotland, UK

**Keywords:** sphingosine-1-phosphate, S1P_1_, apoptosis, Mcl-1, Bim, breast cancer

## Abstract

Although the ability of bioactive lipid sphingosine-1-phosphate (S1P) to positively regulate anti-apoptotic/pro-survival responses by binding to S1P_1_ is well known, the molecular mechanisms remain unclear. Here we demonstrate that expression of S1P_1_ renders CCL39 lung fibroblasts resistant to apoptosis following growth factor withdrawal. Resistance to apoptosis was associated with attenuated accumulation of pro-apoptotic BH3-only protein Bim. However, although blockade of extracellular signal-regulated kinase (ERK) activation could reverse S1P_1_-mediated suppression of Bim accumulation, inhibition of caspase-3 cleavage was unaffected. Instead S1P_1_-mediated inhibition of caspase-3 cleavage was reversed by inhibition of phosphatidylinositol-3-kinase (PI3K) and protein kinase C (PKC), which had no effect on S1P_1_ regulation of Bim. However, S1P_1_ suppression of caspase-3 was associated with increased expression of anti-apoptotic protein Mcl-1, the expression of which was also reduced by inhibition of PI3K and PKC. A role for the induction of Mcl-1 in regulating endogenous S1P receptor-dependent pro-survival responses in human umbilical vein endothelial cells was confirmed using S1P receptor agonist FTY720-phosphate (FTY720P). FTY720P induced a transient accumulation of Mcl-1 that was associated with a delayed onset of caspase-3 cleavage following growth factor withdrawal, whereas Mcl-1 knockdown was sufficient to enhance caspase-3 cleavage even in the presence of FTY720P. Consistent with a pro-survival role of S1P_1_ in disease, analysis of tissue microarrays from ER^+^ breast cancer patients revealed a significant correlation between S1P_1_ expression and tumour cell survival. In these tumours, S1P_1_ expression and cancer cell survival were correlated with increased activation of ERK, but not the PI3K/PKB pathway. In summary, pro-survival/anti-apoptotic signalling from S1P_1_ is intimately linked to its ability to promote the accumulation of pro-survival protein Mcl-1 and downregulation of pro-apoptotic BH3-only protein Bim via distinct signalling pathways. However, the functional importance of each pathway is dependent on the specific cellular context.

D-*erythro*-sphingosine-1-phosphate (S1P) is a bioactive lipid produced in large quantities by several cell types, including erythrocytes and activated platelets.^1^ It has also been found to be an active constituent of high- and low-density lipoproteins and may be responsible for their cytoprotective effects on vascular endothelial cells (ECs).^2^ S1P is generated mainly by hydrolysis of sphingomyelin and the sequential action of ceramidase and sphingosine kinases 1 and 2 (SK1 and 2) on ceramide and sphingosine.^3^ The effects of extracellular S1P are mediated by a family of five G-protein-coupled receptors termed S1P_1_–S1P_5_.^4, 5^

The clinical use of FTY720 (fingolimod), which is phosphorylated by SK2 to form the S1P receptor agonist FTY720-phosphate (FTY720P) for management of relapsing-remitting multiple sclerosis (MS), has demonstrated the utility of targeting the S1P receptor family.^5, 6^ However, maximising the wider utility of S1P_1_-targeted drugs will require a greater understanding of the key processes activated upon receptor modulation.

S1P_1_ couples with the G_i_ family of guanine nucleotide-binding regulatory proteins (G-proteins) to activate multiple intracellular signalling pathways, including the extracellular signal-regulated kinases 1,2 (ERK1,2) and the phosphatidylinositol-3-kinase (PI3K) pathways.^5, 7^ It has also been shown that S1P_1_ in ECs stabilises adherens junctions and inhibits sprouting angiogenesis to maintain nascent blood vessel stability^8, 9^ while S1P_1_ antagonism inhibits tumour vascularisation.^10^ The importance of S1P_1_ in solid tumour progression has also been demonstrated in recent studies that have identified a feed-forward mechanism in which S1P_1_-mediated induction of signal transducer and activator of transcription 3-activating genes, such as pleiotropic cytokine IL-6 and S1P_1_ itself, can drive tumour growth and ultimately metastasis.^11, 12, 13^ However, despite the importance of pro-survival signalling downstream of S1P_1_, the mechanisms responsible remain unclear.

The intrinsic apoptotic pathway is controlled by regulated interactions between three classes of ‘B-cell lymphoma 2' (Bcl-2) proteins: multi-domain pro-apoptotic proteins (e.g., Bcl-2-associated X Protein (Bax), Bcl-2 homologous antagonist/killer (Bak)), pro-survival proteins (e.g., Bcl-2, Bcl-2-like protein 1 (Bcl-X_L_), myeloid cell leukaemia sequence 1 (Mcl-1)) and pro-apoptotic Bcl-2 homology domain 3 (BH3)-only proteins (e.g., Noxa, p53-upregulated modulator of apoptosis (PUMA), Bcl-2-modifying factor (Bmf), Bcl-X_L_/Bcl-2-associated death promoter (Bad) and Bcl-2-interacting mediator of cell death (Bim); reviewed in Chipuk *et al.*^14^ and Giam *et al.*^15^). Although Bax and Bak are restrained by interaction with pro-survival proteins, BH3-only proteins function as sensors of cell stress via their activation in response to pro-apoptotic stimuli.^14, 15^ For example, the ‘extra long' Bim splice variant BimEL is induced following the inhibition of protein kinase B (PKB)-mediated phosphorylation of transcription factor FOXO3A that occurs following growth factor withdrawal, leading to a reduced binding of 14-3-3 proteins, which triggers FOXO3A translocation to the nucleus to initiate Bim transcription.^16^ In addition, ERK1,2-mediated phosphorylation of BimEL promotes its dissociation from pro-survival proteins, such as Mcl-1 and Bcl-X_L_, to enhance cell survival^17^ and also primes BimEL for further phosphorylation by ribosomal S6 kinase, which ultimately promotes BimEL polyubiquitylation and proteasomal degradation.^18^

Despite the well-documented ability of S1P_1_ receptor activation to increase survival in a variety of cell types, the molecular mechanisms responsible have not been fully characterised. In this study, we demonstrate that endogenous and recombinant S1P_1_ is able to suppress levels of pro-apoptotic BH3-only protein Bim via a mitogen-activated protein/ERK kinase (MEK)/ERK1,2 pathway, and enhance the accumulation of anti-apoptotic protein Mcl-1 via a PI3K-mediated and protein kinase C (PKC)-mediated pathway. These findings have clinical relevance, as S1P_1_ expression is associated with enhanced ERK pathway activation and reduced apoptosis in ER^+^ breast cancer tissue. Thus, multiple pathways appear to be critical in determining the ability of S1P_1_ to enhance cell survival.

## Results and Discussion

### S1P_1_ expression enhances survival of CCL39 cells following growth factor withdrawal

**Figure 1:**
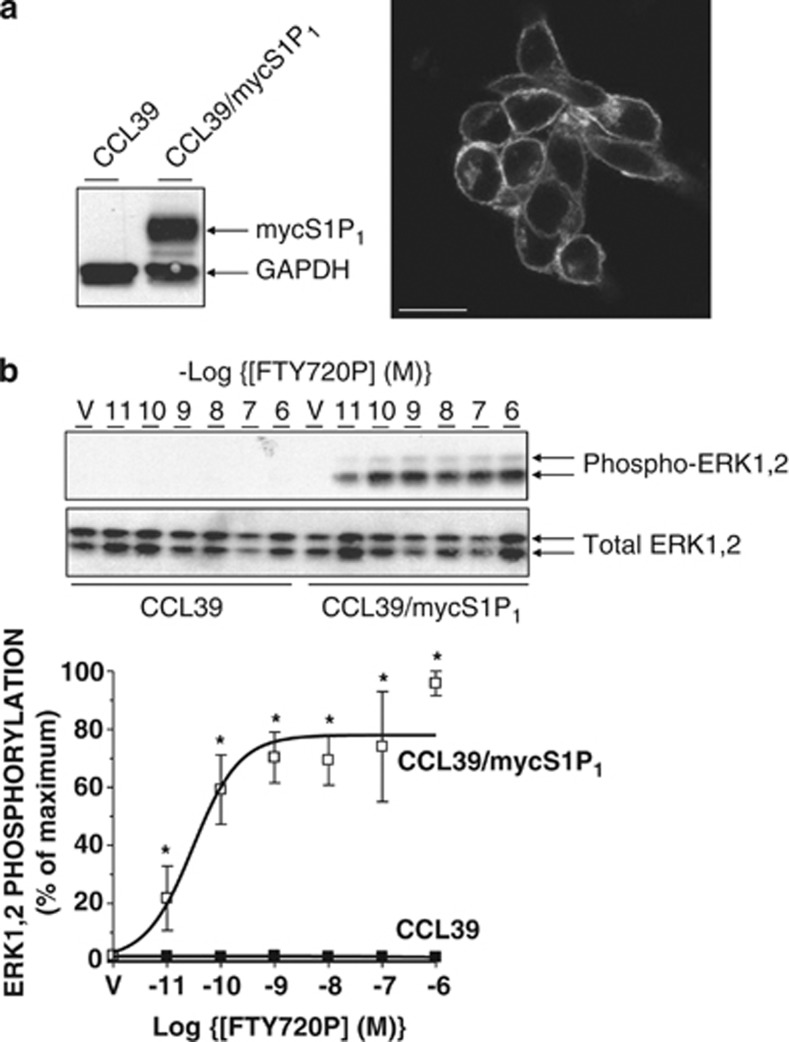
Functional expression of human S1P_1_ in CCL39 cells. (**a**) Left: detergent-soluble cell extracts from control and S1P_1_-expressing CCL39 cells were equalised for protein content before immunoblotting with anti-myc antibody 9E10 (to detect myc epitope-tagged receptor) and GAPDH. Right: S1P_1_-expressing CCL39 cells were fixed and permeabilised for staining with 9E10 antibody and Alexa 488-conjugated goat anti-mouse IgG before visualisation by confocal microscopy. Scale bar=20 microns. (**b**) Upper: control and S1P_1_-expressing CCL39 cells were treated for 5 min with the indicated concentrations of S1P receptor agonist FTY720P or vehicle (V) before the preparation of detergent-soluble cell extracts. Samples were equalised for protein content before fractionation via SDS-PAGE and subsequent immunoblotting with anti-Thr202/Tyr204 phospho-specific ERK1,2 antibodies as a surrogate marker of intracellular signalling. Equal protein loading was assessed by determining total ERK1,2 levels. Lower: data are presented as mean values±S.E. for *n*=3 separate experiments. *P<0.05 *versus* identically treated CCL39 controls

As enhanced survival, or a resistance to apoptosis, is a key aspect of many pathologies, a greater understanding of the mechanisms responsible for S1P_1_-mediated cell survival responses at the molecular level is required to fully exploit the possibility of therapeutically targeting this receptor in disease. To examine S1P_1_ function, we initially utilised CCL39 hamster lung fibroblasts stably expressing a myc epitope-tagged human S1P_1_ receptor (Figure 1a). Receptors were localised to the cell surface (Figure 1a) and no staining was detectable in control CCL39 cells, thus confirming the specificity of the immunostaining for S1P_1_ (data not shown). Treatment of these cells with FTY720P was able to sustain a concentration-dependent activation of the ERK1,2 pathway (Figure 1b) with a pEC_50_=10.52±0.32 (*n*=3). This is consistent with the sub nM affinity of ligand binding to human S1P_1_^19^ and the nM potency reported for activation of downstream signalling at recombinant and endogenous S1P_1_ receptors.^19, 20^ No ERK1,2 activation was detectable in CCL39 neomycin-resistant clones (hereafter referred to as ‘controls') indicating that the response to FTY720P was mediated entirely by S1P_1_.

**Figure 2:**
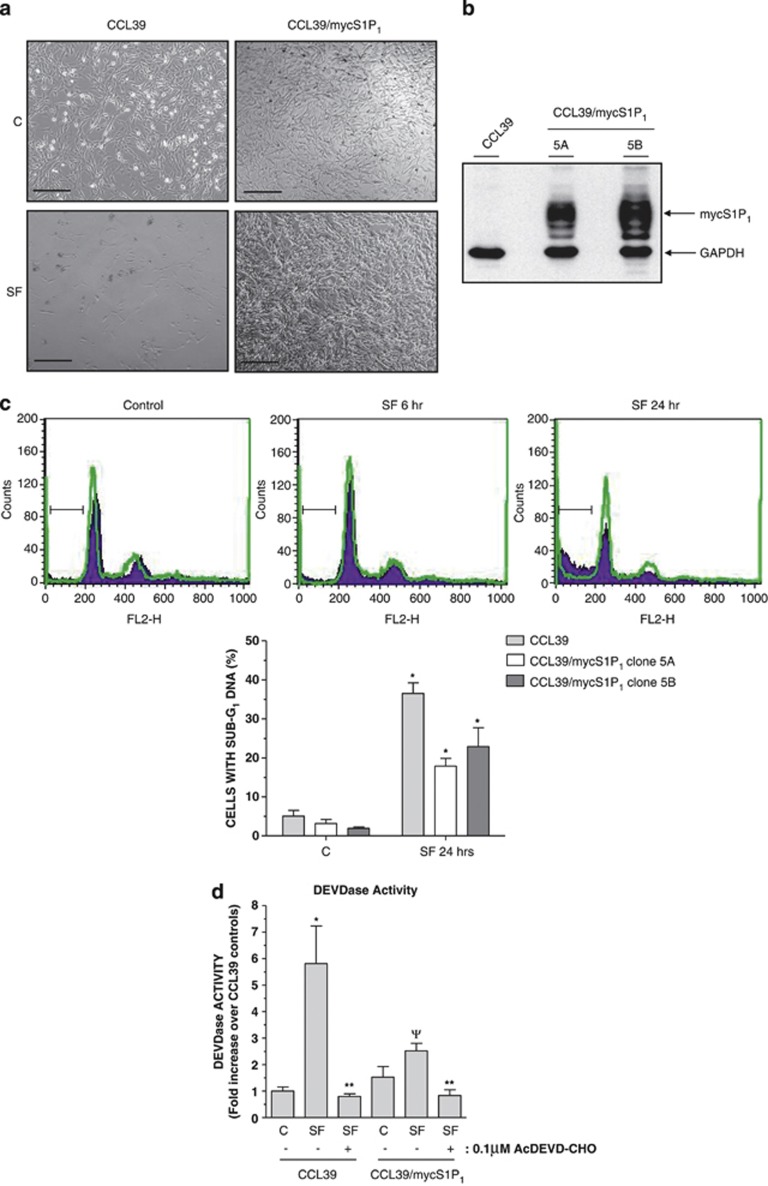
S1P_1_-expressing CCL39 cells are resistant to growth factor withdrawal-induced apoptosis. (**a**) Phase contrast images of control and S1P_1_-expressing CCL39 cells maintained in normal growth medium after switching to either fresh growth medium (C) or serum-free medium (SF) for 24 h. Scale bar=100 microns. (**b**) Detergent-soluble cell extracts from control and S1P_1_-expressing CCL39 cell lines 5A and 5B were equalised for protein content before immunoblotting with anti-myc antibody 9E10 and GAPDH. (**c**) Control and S1P_1_-expressing CCL39 cell lines 5A and 5B maintained in normal growth medium were switched to either serum-free medium or fresh growth medium before fixation and staining with PI and FACS analysis of cell cycle status. Representative traces from control (purple) and clone 5A S1P_1_-expressing (green) CCL39 cells in either control growth medium or serum-free (SF) medium for the indicated times are shown along with the sub-G_1_ gates. Lower panel: data taken from *n*=3 experiments showing the accumulation of sub-G_1_ cells. *P<0.05 *versus* cells grown in control medium, ^Ψ^*P*<0.05 *versus* CCL39 control cells at the indicated time point. (**d**) Control and S1P_1_-expressing CCL39 cell line 5A were switched to either SF medium or fresh growth medium (C) for 16 h before preparation of protein-equalised cell extracts for assay of DEVDase activity using DEVD-*p*NA as substrate in the presence or absence of caspase-3/7 inhibitor AcDEVD-CHO (0.1 *μ*M). Data are presented as mean values±S.E. for *n*=3 separate experiments. *P<0.05 *versus* CCL39 controls in normal growth medium, **P<0.05 *versus* cell extracts in the absence of AcDEVD-CHO, ^Ψ^*P*<0.05 *versus* serum-starved CCL39 controls

The intrinsic apoptotic responses of CCL39 cells upon serum withdrawal have already been well characterised^17, 21, 22^ and therefore provides a useful system for examining the effect of agents on S1P_1_ expression and activation. It was noted that S1P_1_-expressing cells were viable after serum withdrawal for up to 24 h, whereas control CCL39 cells were apoptotic (Figure 2a). FACS analysis of cell cycle distributions following propidium iodide (PI) staining confirmed that while growth factor withdrawal produced a time-dependent increase in the number of control cells with sub-G_1_ DNA, this response was significantly inhibited in the CCL39/mycS1P_1_ cell line we have used for most of our study (cell line 5A) and an additional line (5B) (Figures 2b and c). This suggested that S1P_1_ expression conferred a resistance to serum withdrawal-induced apoptosis in multiple CCL39 cell lines. Cell line 5A was used for the remainder of the experiments presented in this study.

**Figure 3:**
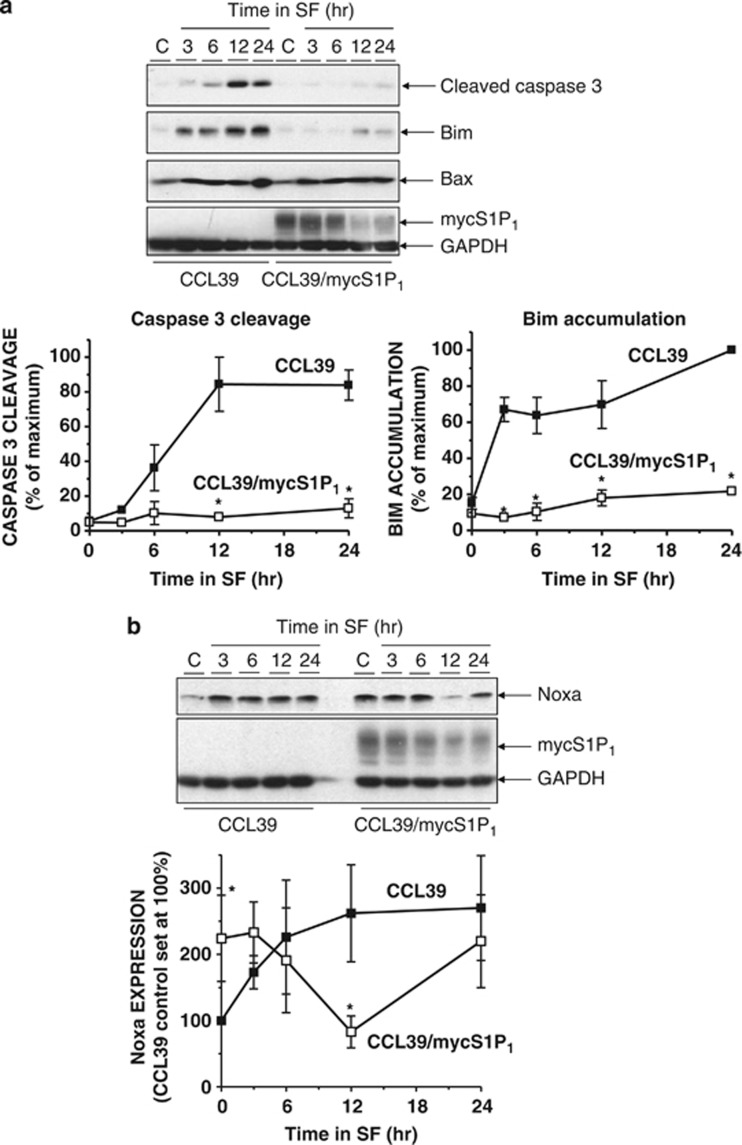
Regulation of pro-apoptotic protein expression in S1P_1_-expressing CCL39 cells. (**a**) Control and S1P_1_-expressing CCL39 cells were switched to serum-free medium (SF) for the indicated times before preparation of detergent-soluble cell extracts. Samples were equalised for protein content before fractionation via SDS-PAGE and subsequent immunoblotting with the indicated antibodies. Quantitation of cleaved caspase-3 and Bim expression normalised to GAPDH is presented as mean values±S.E. for *n*=3 separate experiments. *P<0.05 *versus* CCL39 control cells at the indicated time point. (**b**) Control and S1P_1_-expressing CCL39 cells were switched to SF medium for the indicated times before preparation of detergent-soluble cell extracts. Samples were equalised for protein content before fractionation via SDS-PAGE and subsequent immunoblotting with the indicated antibodies. Quantitation of Noxa expression normalised to GAPDH is presented as mean values±S.E. for *n*=3 separate experiments. *P<0.05 *versus* CCL39 control cells at the indicated time point

As many features of apoptotic cell death are triggered by caspases, we compared the effects of growth factor withdrawal on DEVDase activity in lysates from control and S1P_1_-expressing CCL39 cells. Growth factor removal produced an increase in DEVDase activity in cell lysates that was attenuated by co-incubation with the caspase-3/7 inhibitor Ac.DEVD-CHO (Figure 2d). However, the growth factor withdrawal-induced increase in DEVDase activity in lysates from S1P_1_-expressing cells was significantly reduced compared with control CCL39 cells (Figure 2d). Consistent with these observations, immunoblotting of detergent-soluble cell lysates revealed that serum deprivation of control CCL39 cells resulted in the time-dependent formation of cleaved activated caspase-3, which was detectable at 6 h, maximal at 12 h and sustained for at least 24 h (Figure 3a). However, serum deprivation of S1P_1_-expressing cells had little effect on levels of cleaved caspase-3 (Figure 3a). Consistent with previous studies,^21^ the appearance of cleaved caspase-3 in growth factor-deprived control CCL39 cells was preceded by the accumulation of pro-apoptotic protein Bim. In parallel with the effect on caspase-3 activation, Bim expression following serum withdrawal was reduced in S1P_1_-expressing cells. Interestingly, although expression levels of BH3-only protein Noxa were initially low in control cells, serum deprivation resulted a rapid accumulation of Noxa that was sustained up to the last time point examined (24 h). Moreover, levels of Noxa were high in S1P_1_-expressing cells and only decreased significantly after serum deprivation for 12 h before returning to the levels observed in cells maintained in normal growth medium (Figure 3b). In contrast, levels of Bax were not altered by either S1P_1_ expression or serum withdrawal (Figure 3a). Levels of related BH3-only proteins Bmf and PUMA were undetectable in either control or S1P_1_-expressing cells in the presence or absence of serum (data not shown).

### Pharmacology of S1P_1_ pro-survival response

**Figure 4:**
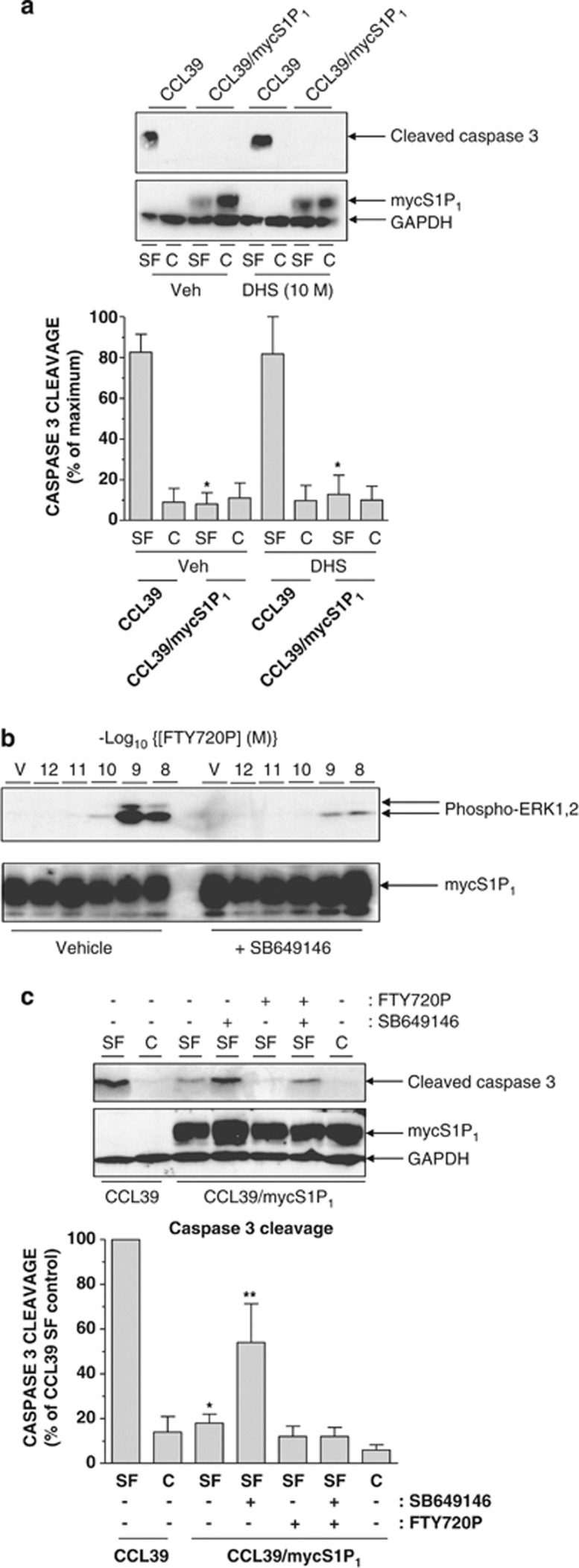
Pharmacology of the S1P_1_-mediated pro-survival response in CCL39 cells. (**a**) Control and S1P_1_-expressing CCL39 cells were switched to either serum-free medium (SF) or fresh growth medium (C) for 16 h in the presence or absence of SK inhibitor DHS (10 *μ*M) before preparation of cell extracts, fractionation via SDS-PAGE and immunoblotting with the indicated antibodies. Quantitation of cleaved caspase-3 levels normalised to GAPDH in control and S1P_1_-expressing CCL39 cells is presented as mean values±S.E. for *n*=3 separate experiments. *P<0.05 *versus* similarly treated CCL39 control cells. (**b**) S1P_1_-expressing CCL39 cells were pre-treated for 15 min with or without SB649146 (5 *μ*M) before treatment for 5 min with the indicated concentrations of S1P receptor agonist FTY720P or vehicle (V) and preparation of detergent-soluble cell extracts. Samples were equalised for protein content before fractionation via SDS-PAGE and subsequent immunoblotting with anti-Thr202/Tyr204 phospho-specific ERK1,2 antibodies and anti-myc antibody 9E10 to detect S1P_1_. (**c**) Control and S1P_1_-expressing CCL39 cells were switched to either SF medium or fresh growth medium (C) for 16 h in the presence or absence of high affinity S1P receptor agonist FTY720P (0.1 *μ*M) or SB649146 (5 *μ*M) either alone or in combination before preparation of cell extracts, fractionation via SDS-PAGE and subsequent immunoblotting with the indicated antibodies. Quantitation of cleaved caspase-3 normalised to GAPDH in control and S1P_1_-expressing CCL39 cells is presented as mean values±S.E. for *n*=3 separate experiments. *P<0.05 *versus* similarly treated CCL39 control cells, **P<0.05 *versus* SF-treated CCL39/mycS1P1 cells treated with vehicle

First, we determined whether the pro-survival effect of S1P_1_ expression was due to formation of endogenous S1P.^23^ However, incubation with pan-SK inhibitor D,L-*threo*-dihydrosphingosine (DHS)^24^ (10 *μ*M) failed to reduce the ability of S1P_1_ to suppress cleaved caspase-3 formation (Figure 4a). Thus, resistance to apoptosis appears to be due to the action of constitutively activated S1P_1_ rather than autocrine production of S1P. To further examine the pharmacology of the response, we utilised FTY720P and S1P_1_-modifying agent SB649146, which has inverse agonist activity against constitutive S1P_1_ receptor-dependent activation of G_i_ and also blocks S1P-dependent activation of ERK1,2.^25, 26, 27^ Treatment of cells with SB649146 (5 *μ*M) reduced FTY720P-stimulated ERK1,2 phosphorylation in S1P_1_-expressing CCL39 cells (Figure 4b: SB649146 reduced 1 nM FTY720P-stimulated ERK1,2 phosphorylation by 75±11%, *n*=4 experiments, *P*<0.05). Treatment of S1P_1_-expressing cells with FTY720P (0.1 *μ*M) during growth factor withdrawal did not further suppress caspase-3 cleavage over that observed in serum-starved cells (Figure 4c) as this response is maximal with growth factor withdrawal. However, treatment of S1P_1_-expressing cells with SB649146 alone significantly increased caspase-3 cleavage following serum withdrawal compared with vehicle-treated cells (Figure 4c), suggesting that SB649146 inhibits the ability of S1P_1_ to constitutively limit apoptosis. The suppression of caspase-3 cleavage achieved by a combination of serum withdrawal and FTY720P was only partially reversed by SB649146 and this response was not consistently observed. Indeed, FTY720P should compete and diminish the effect of SB649146 and this was observed, thereby confirming that both compounds are acting on S1P_1_.

### S1P_1_ regulation of pro-apoptotic protein expression

Caspase-3 cleavage is the final step in the intrinsic apoptotic pathway typically triggered upon accumulation and/or activation of pro-apoptotic BH3-only proteins.^14, 15^ As Bim accumulation most closely paralleled the accumulation of cleaved caspase-3 in control and S1P_1_-expressing CCL39 cells following serum withdrawal (Figure 3a), we examined any potential relationship between the two phenomena. Initially, we compared the ability of a panel of signalling pathway inhibitors to block the suppressive effects of S1P_1_ expression on cleaved caspase-3 and Bim accumulation following growth factor withdrawal.

**Figure 5:**
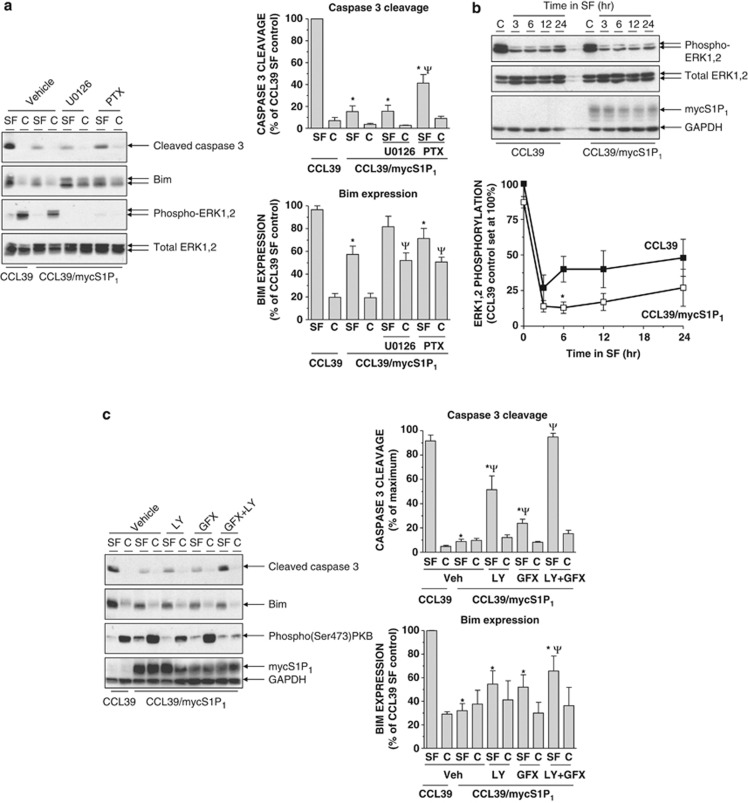
Divergent regulation of Bim accumulation and caspase-3 cleavage in S1P_1_-expressing cells following serum withdrawal. (**a**) Control and S1P_1_-expressing CCL39 cells were switched to either serum-free medium (SF) or fresh growth medium (C) for 16 h in the presence or absence of MEK1,2 inhibitor U0126 (10 *μ*M), pertussis toxin (PTX, 100 ng/ml) or vehicle control for 16 h before preparation of cell extracts, fractionation via SDS-PAGE and subsequent immunoblotting with the indicated antibodies. Quantitation of cleaved caspase-3 and Bim expression normalised to GAPDH in control and S1P_1_-expressing CCL39 cells is presented as mean values±S.E. for *n*=3 separate experiments. *P<0.05 *versus* serum-starved CCL39 control cells, ^Ψ^*P*<0.05 *versus* vehicle-treated CCL39/mycS1P_1_ cells. (**b**) Control and S1P_1_-expressing CCL39 cells were switched to SF medium for the indicated times before preparation of detergent-soluble cell extracts. Samples were equalised for protein content before fractionation via SDS-PAGE and subsequent immunoblotting with the indicated antibodies. Quantitation of phospho-ERK1,2 levels normalised to total ERK1,2 is presented as mean values±S.E. for *n*=3 separate experiments. *P<0.05 *versus* CCL39 control cells at the indicated time point. (**c**) Control and S1P_1_-expressing CCL39 cells were switched to either SF medium or fresh growth medium (C) for 16 h in the presence of PI3K inhibitor LY294002 (LY, 20 *μ*M) or PKC inhibitor GF109203X (5 *μ*M) either alone or in combination, or a vehicle control for 16 h before preparation of cell extracts, fractionation via SDS-PAGE and subsequent immunoblotting with the indicated antibodies. Quantitation of cleaved caspase-3 and Bim expression normalised to GAPDH in control and S1P_1_-expressing CCL39 cells is presented as mean values±S.E. for *n*=3 separate experiments. *P<0.05 *versus* serum-starved CCL39 control cells, ^Ψ^*P*<0.05 *versus* vehicle-treated CCL39/mycS1P_1_ cells

S1P_1_ couples with multiple signalling pathways predominantly via activation of G_i_ proteins.^5, 7^ However, despite almost abolishing ERK1,2 phosphorylation, inactivation of G_i_ proteins with pertussis toxin did not completely reverse S1P_1_-mediated suppression of caspase-3 cleavage, suggesting that S1P_1_-mediated protection against caspase-3 activation is only partially G_i_ dependent (Figure 5a).

Consistent with previous work in CCL39 cells,^17, 21^ treatment of S1P_1_-expressing cells with MAP/ERK kinase (MEK) inhibitor U0126 during serum starvation abolished ERK1,2 phosphorylation and restored Bim expression to levels comparable with those in serum-starved control cells (Figure 5a). This would be consistent with the reported ability of Bim to serve as a substrate for activated ERK1,2, an event that precedes its dissociation from pro-survival proteins and proteasomal degradation.^17, 18, 28^ However, the ability of S1P_1_-expressing cells to limit the generation of cleaved caspase-3 was not altered by U0126 under conditions in which it abolished ERK1,2 phosphorylation (Figure 5a). Interestingly, despite significant suppression of Bim expression in S1P_1_-expressing cells, levels of phosphorylated ERK1,2 in serum-starved S1P_1_-expressing cells *versus* controls were comparable. Detailed analysis of changes in phospho-ERK1,2 levels following serum withdrawal in control and S1P_1_-expressing cells revealed that the decline in phospho-ERK1,2 levels observed was marginally greater in S1P_1_-expressing cells, with the difference reaching statistical significance at the 12-h time point (Figure 5b). As the S1P_1_-mediated suppression of Bim is clearly MEK/ERK dependent (Figure 5a) and thus consistent with previous observations,^17, 18, 21, 28^ our data would argue that S1P_1_ expression must trigger constitutive activation of a localised pool of ERK in CCL39 cells that, although it comprises a small proportion of the total ERK content, is essential for suppression of Bim. The existence of functionally discrete pools of S1P_1_ receptors has been shown in murine embryonic fibroblasts, airway smooth muscle cells and transfected HEK293 cells.^26, 29^ It is also consistent with observations of activation of distinct subcellular pools of ERK1,2 by G-protein-coupled receptors via both G-protein- and *β*-arrestin-mediated processes.^30, 31^

S1P_1_ is also known to activate PKB and conventional and novel PKC isoforms^32, 33^ and we found that levels of active Ser 473-phosphorylated PKB were elevated in S1P_1_-expressing cells either in the presence or absence of serum (Figure 5c). As these are involved in pro-survival responses (reviewed in refs Zhang *et al.*^34^ and Griner and Kazanietz^35^), we examined the effects of inhibiting these pathways individually and in combination. Treatment of cells with conventional and novel PKC inhibitor GF109203X produced a small but significant reversal in the ability of S1P_1_ to limit caspase-3 cleavage. The effect of PI3K inhibitor LY294002 was greater than that of GF109203X. However, in combination both compounds completely reversed the effect of S1P_1_ expression and resulted in cleavage of caspase-3 to a level comparable with that observed in serum-deprived control CCL39 cells (Figure 5c). Importantly, treatment with GF109203X and LY294002 alone did not change the effect of serum withdrawal on Bim accumulation, whereas the presence of both compounds produced only a small increase in Bim, which did not reach the levels observed in control cells (Figure 5c).

### S1P_1_ specifically regulates levels of anti-apoptotic protein Mcl-1

Previous studies have demonstrated a role for Bim in triggering apoptosis in CCL39 cells following serum deprivation.^17, 21^ However, although S1P_1_ could suppress Bim accumulation, restoration of Bim expression by treatment with MEK inhibitor U0126 was not sufficient to increase caspase-3 cleavage. Thus, the pro-survival effect of S1P_1_ was independent of its ability to suppress Bim, which suggested that S1P_1_ must utilise additional mechanisms to maintain cellular resistance to apoptosis even when Bim levels are elevated. One possibility was that S1P_1_ could induce the expression of anti-apoptotic/pro-survival proteins capable of inhibiting Bim. Candidates included Mcl-1, Bcl-X_L_ and Bcl-2, which can sequester BH3-only proteins as well as pro-apoptotic mediators such as Bax and Bak.^14, 15^ Moreover, forced expression of either Mcl-1 or Bcl-2 has been shown to reduce levels of sub-G1/apoptotic CCL39 cells following growth factor withdrawal.^17^ Indeed, the loss of ‘phosphatase and tensin homologue on chromosome 10' expression in murine embryonic fibroblasts can protect against serum withdrawal-induced apoptosis via upregulation of Mcl-1.^36^

**Figure 6:**
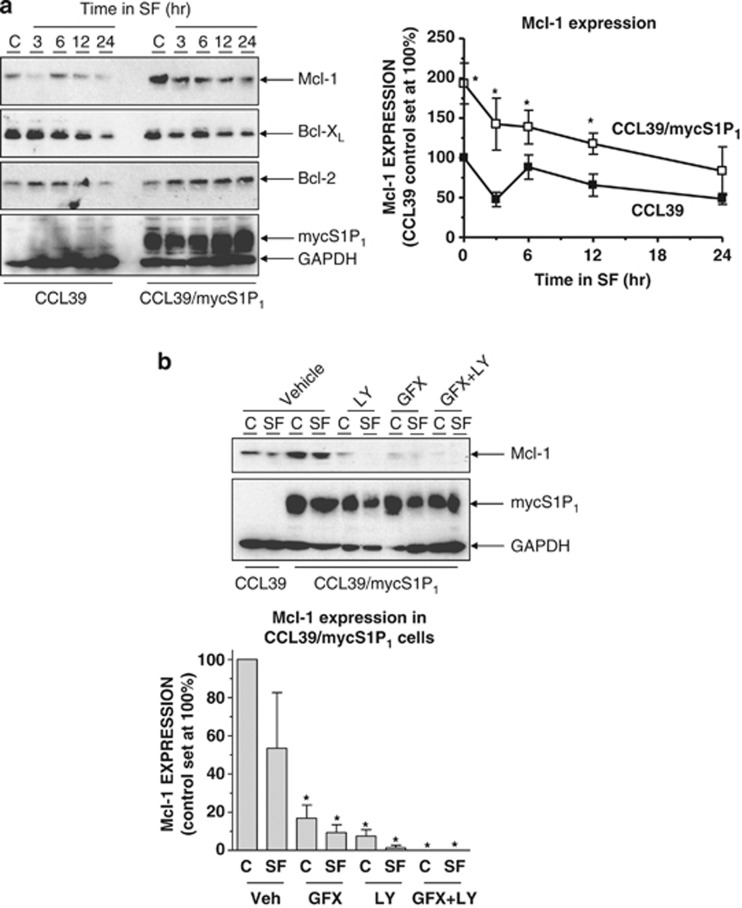
S1P_1_ regulates expression of pro-survival protein Mcl-1. (**a**) Control and S1P_1_-expressing CCL39 cells were switched to serum-free medium (SF) for the indicated times before preparation of detergent-soluble cell extracts. Samples were equalised for protein content before fractionation via SDS-PAGE and subsequent immunoblotting with the indicated antibodies. Quantitation of Mcl-1 expression normalised to GAPDH in control and S1P_1_-expressing CCL39 cells is presented as mean values±S.E. for *n*=3 separate experiments. *P<0.05 *versus* CCL39 control cells at the indicated time point. (**b**) Control and S1P_1_-expressing CCL39 cells were switched to either SF medium or fresh growth medium (C) for 16 h in the presence of LY294002 (LY, 20 *μ*M) or GF109203X (5 *μ*M) either alone or in combination, or a vehicle control for 16 h before preparation of cell extracts, fractionation via SDS-PAGE and subsequent immunoblotting with the indicated antibodies. Quantitation of Mcl-1 expression normalised to GAPDH in control and S1P_1_-expressing CCL39 cells is presented as mean values±S.E. for *n*=3 separate experiments. *P<0.05 *versus* CCL39 control cells, ^Ψ^*P*<0.05 *versus* vehicle-treated CCL39/mycS1P_1_ cells

Therefore, we examined Mcl-1, Bcl-X_L_ and Bcl-2 expression over the same time frame in which differences in cleaved caspase-3 accumulation were apparent. In control CCL39 cells, serum withdrawal produced a time-dependent decrease in Mcl-1; levels were approximately 50% of those for cells grown in serum at 24 h (Figure 6a). Interestingly, Mcl-1 levels were elevated by approximately twofold in S1P_1_-expressing cells in normal growth medium *versus* control CCL39 cells and remained so during growth factor withdrawal up to 24 h (Figure 6a). In contrast, Bcl-2 and Bcl-X_L_ expression levels were comparable at all times after serum withdrawal (Figure 6a).

Several aspects of Mcl-1 regulation also suggested its possible involvement as a mediator of cell survival downstream of S1P_1_. First, Mcl-1 can be degraded by the proteasome after polyubiquitylation by the SCF^FBW7^ E3 ubiquitin ligase complex following phosphorylation by glycogen synthase kinase 3 (GSK3).^37, 38^ Consequently, Mcl-1 accumulation following PKB-mediated phosphorylation and inhibition of GSK3 is an important pro-survival signal.^38^ PKC isoforms have also been shown to regulate Mcl-1 expression.^39^ Given that suppression of caspase-3 activation by S1P_1_ was blocked by PI3K and PKC inhibitors (Figure 5c), we examined their effects on Mcl-1 expression in S1P_1_-expressing cells in the presence and absence of serum. Although Mcl-1 levels were elevated in S1P_1_-expressing cells, treatment with each inhibitor either alone or in combination elicited significant decreases in Mcl-1 expression (Figure 6b) that paralleled the observed changes in caspase-3 activation (Figure 5c). Therefore, S1P_1_ induces Mcl-1 expression via PI3K- and PKC-dependent pathways and this contributes to the enhanced survival of S1P_1_-expressing cells upon serum withdrawal.

**Figure 7:**
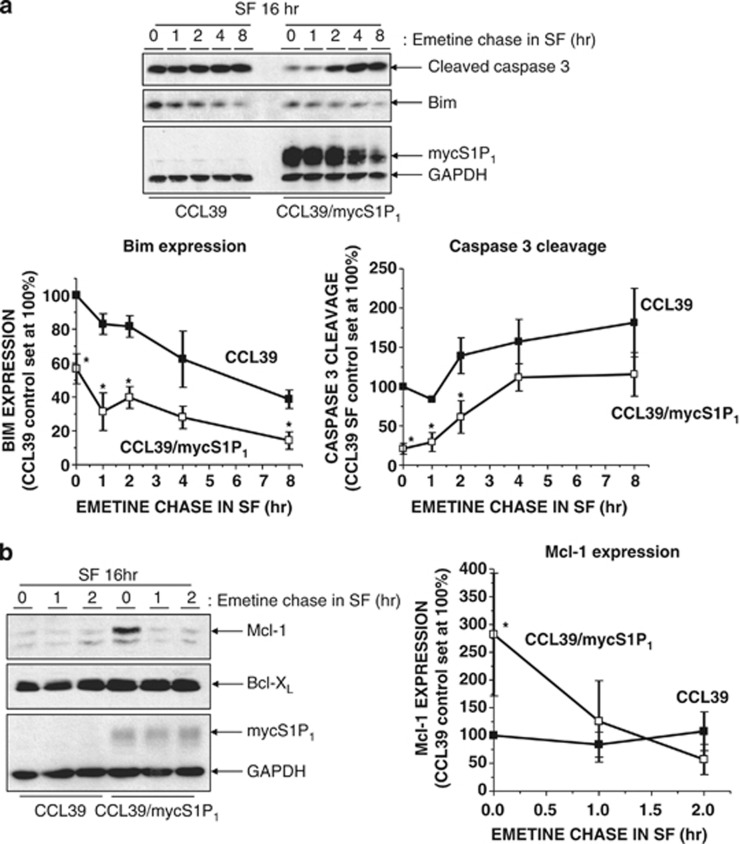
The enhanced survival capacity of S1P_1_-expressing cells requires new protein synthesis and is lost upon Mcl-1 downregulation. (**a**) Control and S1P_1_-expressing CCL39 cells were switched to serum-free medium (SF) for 16 h before washing and incubation in SF for the indicated times in the presence of protein synthesis inhibitor emetine (100 *μ*M). Cell extracts were then prepared for fractionation via SDS-PAGE and subsequent immunoblotting with the indicated antibodies. Quantitation of cleaved caspase-3 and Bim expression normalised to GAPDH in control and S1P_1_-expressing CCL39 cells is presented as mean values±S.E. for *n*=3 separate experiments. *P<0.05 *versus* CCL39 control cells. (**b**) Control and S1P_1_-expressing CCL39 cells were switched to SF medium for 16 h before washing and incubation in SF for the indicated times in the presence of protein synthesis inhibitor emetine as described in (**a**). Cell extracts were then prepared for fractionation via SDS-PAGE and subsequent immunoblotting with the indicated antibodies. Quantitation of Mcl-1expression normalised to GAPDH in control and S1P_1_-expressing CCL39 cells is presented as mean values±S.E. for *n*=3 separate experiments. *P<0.05 *versus* CCL39 control cells

To further examine a link between elevated Mcl-1 expression and resistance to apoptosis in S1P_1_-expressing cells, we tested the effects of inhibiting new protein synthesis on caspase-3 activation in control and S1P_1_-expressing CCL39 cells. When serum-starved cells were switched to serum-free medium containing protein synthesis inhibitor emetine, cleaved caspase-3 levels in control CCL39 cells remained consistently elevated for up to 8 h (Figure 7a). In contrast, the initially low levels of cleaved caspase-3 found in S1P_1_-expressing cells increased until, by 4 h, they were similar to those observed in control cells. Moreover, this occurred despite parallel decreases in pro-apoptotic Bim expression in both cell lines (Figure 7a). To determine whether changes in Mcl-1 expression could explain increased caspase-3 activation in S1P_1_-expressing cells following emetine treatment, we compared Mcl-1 levels in control and S1P_1_-expressing cells. Interestingly, although Mcl-1 protein levels remained relatively stable in serum-starved control CCL39 cells following emetine treatment, levels in S1P_1_-expressing cells were dramatically reduced within 2 h despite being expressed at higher levels initially than control cells. In contrast, levels of related anti-apoptotic protein Bcl-X_L_ remained constant (Figure 7b). Importantly, the rapid downregulation of Mcl-1 preceded the increase in cleaved caspase-3 observed in S1P_1_-expressing cells (Figures 7a and b), suggesting that maintenance of Mcl-1 expression prevents caspase-3 activation thereby conferring resistance to apoptosis.

### Mcl-1 and endogenous S1P receptor regulation of apoptosis in vascular ECs

**Figure 8:**
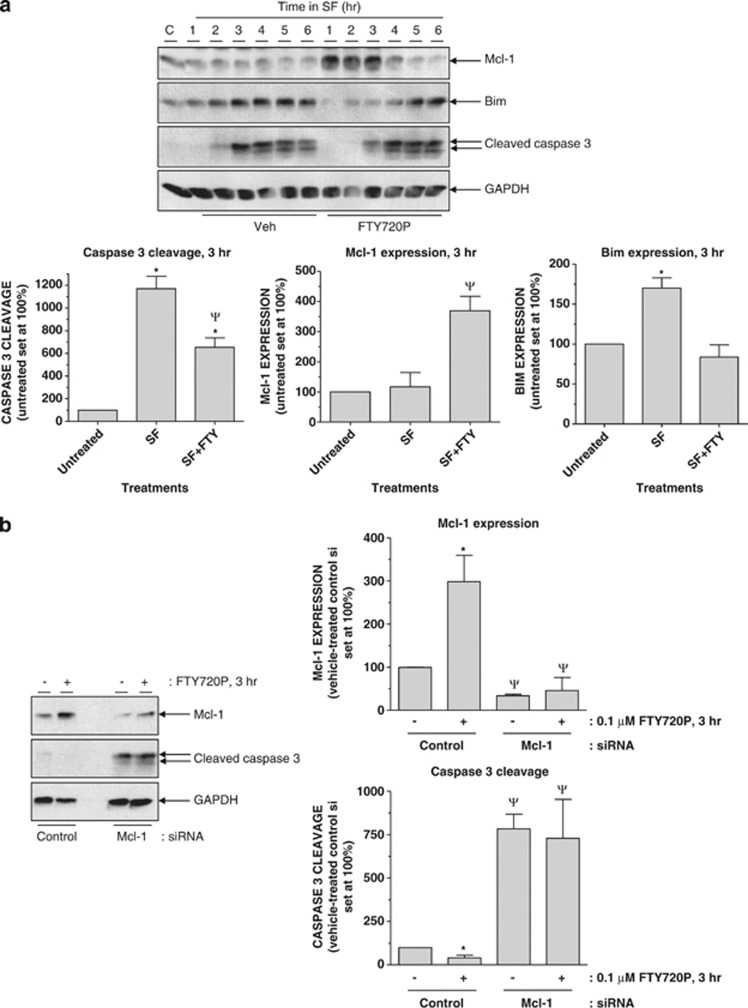
Activation of endogenous S1P receptors by FTY720P induces Mcl-1 to delay the onset of apoptosis in vascular ECs following growth factor withdrawal. (**a**) HUVECs were switched to serum- and growth factor-free medium in the presence or absence of FTY720P (0.1 *μ*M) for the indicated times before preparation of detergent-soluble cell extracts. Samples were equalised for protein content and fractionated via SDS-PAGE for subsequent immunoblotting with the indicated antibodies. Quantitation of caspase-3 cleavage, Bim expression and Mcl-1 expression at the indicated times normalised to GAPDH is presented as mean values±S.E. for *n*=3 separate experiments. *P<0.05 *versus* cells maintained in normal growth medium, ^Ψ^*P*<0.05 *versus* growth factor-deprived cells treated with vehicle. (**b**) HUVECs were switched to serum- and growth factor-free medium in the presence or absence of FTY720P (0.1 *μ*M) for 3 h before preparation of detergent-soluble cell extracts. Samples were equalised for protein content and fractionated via SDS-PAGE for subsequent immunoblotting with the indicated antibodies. Quantitation of caspase-3 cleavage and Mcl-1 expression normalised to GAPDH is presented as mean values±S.E. for *n*=3 separate experiments. *P<0.05 *versus* cells treated without FTY720P at the same time point, ^Ψ^*P*<0.05 *versus* corresponding control siRNA-treated samples

To examine the potential significance of Mcl-1 regulation in cells expressing endogenous S1P_1_, we utilised human umbilical vein ECs (HUVECs) as several studies have demonstrated S1P_1_ activation of PKC and PI3K pathways in these cells.^32, 33, 40^ Growth factor removal resulted in a time-dependent increase in levels of cleaved caspase-3 that peaked at 3 h and was sustained for up to 6 h (Figure 8a). Inclusion of S1P receptor agonist FTY720P triggered a rapid yet transient accumulation of Mcl-1, which accompanied a delayed accumulation of Bim and cleaved caspase-3 *versus* vehicle-treated controls (Figure 8a). To examine the role of Mcl-1 accumulation in repressing caspase-3 cleavage, we tested the effects of siRNA-mediated Mcl-1 knockdown. Importantly, the ability of FTY720P to increase Mcl-1 expression was significantly attenuated in Mcl-1 siRNA- *versus* non-targeting control siRNA-transfected HUVECs. This resulted in a significant increase in cleaved caspase-3 levels and blocked the ability of FTY720P to inhibit caspase-3 activation (Figure 8b).

### S1P_1_ expression and resistance to apoptosis in oestrogen receptor-positive (ER^+^) breast cancer

The ability to evade apoptosis is one of the hallmarks of cancer^41^ and increased expression of pro-survival proteins can promote resistance of mammary tumours to chemo- and radiotherapies.^42, 43^ As enhanced plasma membrane expression of S1P_1_ in ER^+^ breast cancer is associated with poor prognosis in patients treated with tamoxifen,^44^ we examined the relationship between S1P_1_ expression and apoptotic status in tissue microarrays (TMAs) from a previously described cohort of ER^+^ breast cancer patients.^45^

**Figure 9:**
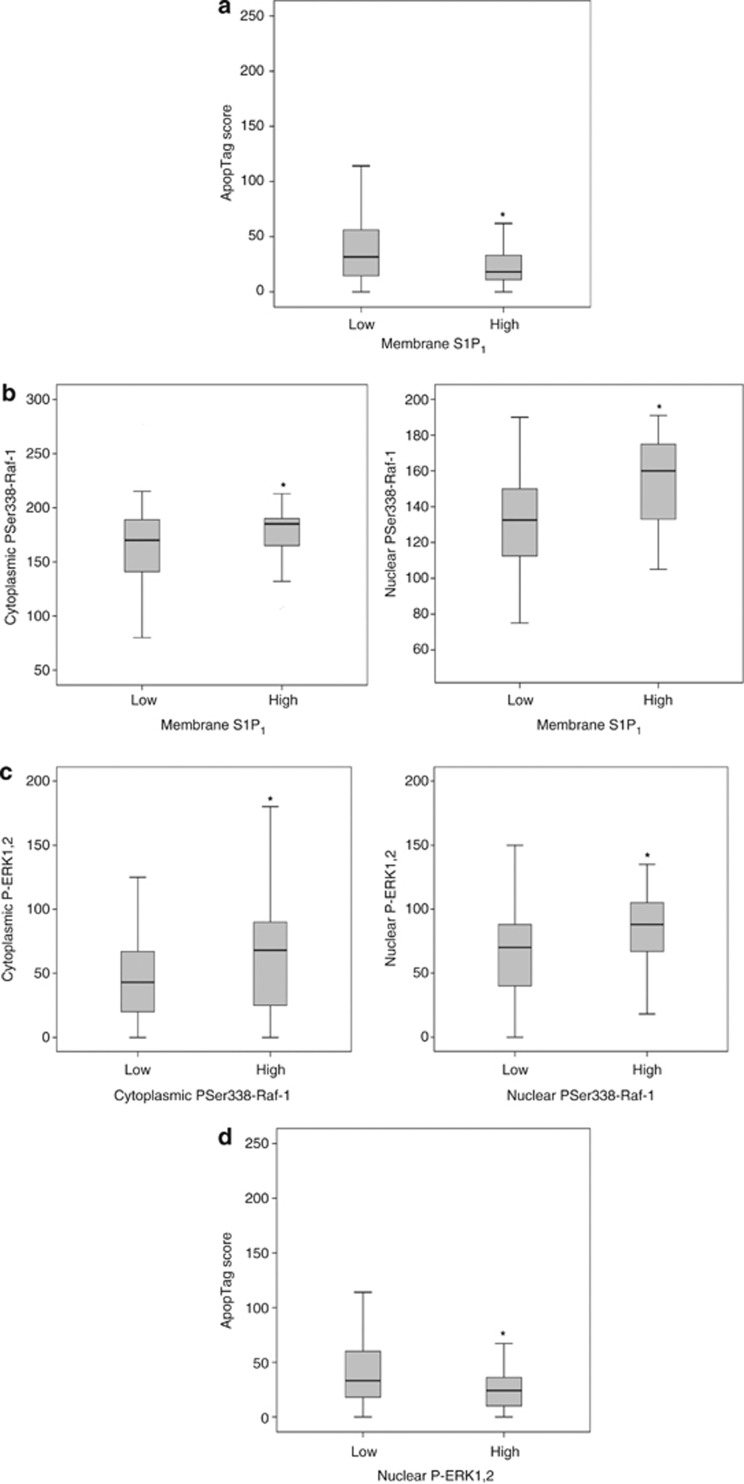
Correlations between elevated S1P_1_ expression, ERK1,2 pathway activation and enhanced survival in ER^+^ breast cancer tumours. (**a**) High levels of plasma membrane-localised S1P_1_ expression correlate significantly with a low apoptosis score (*P*=0.044). (**b**) High levels of plasma membrane-localised S1P_1_ expression are significantly associated with elevated levels of both cytoplasmic (*P*=0.038) and nuclear (*P*=0.0001) active phospho-Ser338 Raf-1. (**c**) High levels of phosphorylated ERK1,2 are significantly associated with elevated levels of active phospho-Ser338 Raf-1 in both the cytoplasm (*P*=0.004) and nucleus (*P*=0.007). (**d**) High levels of phosphorylated ERK1,2 in the nucleus are significantly associated with a low apoptosis score (*P*=0.012)

Our experiments in CCL39 cells and HUVECs suggested that S1P_1_ could potentially enhance cell survival via either a MEK/ERK1,2-dependent suppression of BH3-only protein Bim expression or a PI3K- and PKC-mediated accumulation of pro-survival protein Mcl-1. Consistent with a pro-survival role of S1P_1_ in ER^+^ breast cancer, tumours with high levels of S1P_1_ expression in the plasma membrane were correlated with significantly lower levels of apoptosis-derived DNA fragments in TMAs (Figure 9a). Analysis of candidate S1P_1_-activated signalling pathways revealed a significant association between high levels of S1P_1_ expression in the plasma membrane and elevated levels of Ser338-phosphorylated active Raf-1 in cytoplasmic and nuclear compartments (Figure 9b). As might be expected, high levels of Ser338-phosphorylated active Raf-1 in the cytoplasm and nucleus were associated with elevated levels of Thr202/Tyr204 phosphorylated active ERK1,2 in both compartments (Figure 9c). Importantly, high levels of phosphorylated active ERK1,2 were also associated with a significantly lower apoptosis-derived DNA fragments in tumour samples (Figure 9d), whereas no significant association between low apoptosis scores and enhanced phosphorylation of PKB was observed in this cohort of patients (data not shown). Although these data are essentially correlative and not necessarily indicative of a causal relationship, taken together they would argue that while S1P_1_ can initiate multiple intracellular pro-survival signals, elevated levels of S1P_1_ in ER^+^ breast tumours might suppress apoptotic cell death predominantly via a Raf-1/MEK/ERK1,2 pathway.

In summary, we have identified multiple mechanisms by which S1P_1_ expression and activation can enhance cell survival. First, we have demonstrated a critical role for Mcl-1 in enhancing cell survival following growth factor withdrawal in two cell models. Several studies have demonstrated elevated Mcl-1 in different cancer settings, including multiple myeloma^46^ and hepatocellular carcinoma,^47^ and its increased expression may underlie the resistance of some tumours to BH3 mimetic drug ABT-737, which binds Mcl-1 relatively poorly compared with other anti-apoptotic Bcl-2 family members.^48, 49^ In addition, the importance of Mcl-1 in promoting the EC survival necessary for angiogenesis has been demonstrated by studies showing that its downregulation in ECs contributes to the pro-apoptotic and anti-angiogenic effects of 3,6-di(2,3-epoxypropoxy)xanthone and sorafenib.^50, 51^ Therefore, the ability of S1P_1_ to enhance Mcl-1 expression may have an important role in the control of EC viability and stabilisation of mature vasculature by this receptor.^8, 9^

Second, we have shown that in ER^+^ breast cancers, high S1P_1_ expression and resistance to apoptosis is linked to hyperactivation of the ERK1,2 pathway, which is a key suppressor of pro-apoptotic BH3-only protein Bim. Therefore, it might be beneficial to target these pathways to treat ER^+^ breast cancers for which significant correlations between poor prognosis and high expression levels of SK1, which catalyses formation of S1P, and the S1P receptors S1P_1_ and S1P_3_ have been reported for specific patient cohorts.^45^ Our delineation of distinct ERK1,2- and PI3K/PKC-dependent processes downstream of S1P_1_ also suggest that S1P_1_-selective antagonists may be useful in attenuating multiple pro-survival pathways not only in tumour cells but also in ECs within the tumour vasculature. Indeed, it has recently been demonstrated that the SK-phosphorylated product of S1PR antagonist pro-drug VPC03090 inhibits mammary tumour growth in mice,^52^ further supporting the hypothesis that S1PRs are potentially efficacious therapeutic targets for developing new breast cancer treatments.

## Materials and methods

### Materials

FTY720P was obtained from Caymen Chemical (Ann Arbor, MI, USA). Antibodies were from the following sources: Bim (Chemicon, Temecula, CA, USA; cat. no. AB17003), Mcl-1 (Rockland, Gilbertsville, PA, USA; cat. no. 600-401-394), cleaved caspase-3 (Asp175; Cell Signaling Technology, Danvers, MA, USA; cat. no. 9661), Ser473 phospho-specific PKB (Cell Signaling Technology, cat. no. 9271), total ERK1,2 (Cell Signaling Technology, cat. no. 9102), Thr202/Tyr204 phospho-specific ERK1,2 (Cell Signaling Technology, cat. no. 9106), Bax (N-20; Santa Cruz Biotechnology, Dallas, TX, USA; cat. no. sc-493), Bcl-2 (C-2; Santa Cruz Biotechnology, cat. no. sc-7382), Noxa (FL-54; Santa Cruz Biotechnology, cat. no. sc-30209). DEVDase assay reagents were from Chemicon. Non-targeting control siRNA (cat. no. D-001810-10-05) was from Dharmacon (Loughborough, UK) and human Mcl-1-targeted siRNA (cat. no. SI02781205) was from Qiagen (Valencia, CA, USA). Signalling pathway inhibitors (Ac.DEVD-CHO, U0126, LY294002, GF109203X, pertussis toxin) were from Merck Chemicals (Feltham, UK). PI was purchased from Sigma (Gillingham, UK). Sources of all other materials have been described elsewhere.^53, 54, 55^

### Cell culture

CCL39 cells were maintained in Dulbecco's modified Eagle's medium supplemented with 10% (v/v) fetal bovine serum, 1 mM L-glutamine, 100 units/ml penicillin and 100 *μ*g/ml streptomycin at 37 °C in a humidified atmosphere containing 5% (v/v) CO_2_. Stably transfected clones were cultured in medium supplemented with 0.6 mg/ml G418 to maintain selection pressure. HUVECs were propagated in endothelial growth medium-2 (EGM-2) supplemented with 2% (w/v) fetal bovine serum, hydrocortisone, ascorbate and recombinant growth factors as recommended by the supplier (Lonza, Verviers, Belgium).

### Immunofluorescence and confocal microscopy

S1P_1_-expressing CCL39 cells were plated onto glass coverslips in six-well dishes and grown to confluence. Following serum starvation for 16–24 h and fixed by a 20-min incubation at room temperature with 4% (w/v) paraformaldehyde in 5% (w/v) sucrose-phosphate-buffered saline (PBS). Cells were permeabilised in 0.4% (v/v) Triton X-100 in PBS before sequential incubation with rabbit polyclonal anti-S1P_1_ antibody and Alexa 488-conjugated goat anti-rabbit IgG. Coverslips were then mounted onto slides for imaging by confocal microscopy as described previously.^55^

### FACS analysis of cell cycle progression

Treated control or S1P_1_-expressing CCL39 cells were harvested by trypsinisation before re-suspension in ice-cold 70% (v/v) ethanol in PBS and incubation on ice for 30 min and storage at −20 °C. Following brief centrifugation, cells were washed twice in ice-cold PBS then resuspended in PBS (0.5 ml) containing 0.1% (v/v) Triton X-100, RNase A (0.2 mg/ml) and PI (20 *μ*g/ml) for 30 min at 37 °C before flow cytometry on a BD FACSCalibur and analysis using CellQuest Pro (BD Biosciences, Oxford, UK).

### Immunoblotting

Confluent cells in six-well plates were treated as described in the figures before washing in ice-cold PBS and solubilisation by scraping into 0.2 ml per well detergent lysis buffer (50 mM sodium HEPES, pH 7.5, 150 mM sodium chloride, 5 mM EDTA, 10 mM sodium fluoride, 10 mM sodium phosphate, 1% (v/v) Triton X-100, 0.5% (w/v) sodium deoxycholate, 0.1% (w/v) SDS, 0.1 mM phenylmethylsulphonyl fluoride, 10 *μ*g/ml soybean trypsin inhibitor, 10 *μ*g/ml benzamidine and EDTA-free complete protease inhibitor mix). Following brief vortexing and solubilisation by rotation for 30 min, insoluble material was removed by microcentrifugation and the supernatant assayed for protein content using a bicinchonic acid assay. Samples equalised for protein content (typically 10–20 *μ*g/sample) were fractionated by SDS-PAGE on 10 or 12% (w/v) resolving gels. Following transfer to nitrocellulose, membranes were blocked for 1 h at room temperature in blocking buffer (5% (w/v) skimmed milk in PBS containing 0.1% (v/v) Tween-20). Membranes were then incubated either overnight at 4 °C or for 1 h at room temperature with primary antibody diluted in fresh blocking buffer. Primary antibodies were each used at a final concentration of 1 *μ*g/ml. Following three washes in blocking buffer, membranes were incubated for 1 h at room temperature with appropriate horseradish peroxidase-conjugated secondary antibody at a 1 in 1000 dilution. After further washes with blocking buffer and PBS, immunoreactive proteins were visualised by enhanced chemiluminescence. For phospho-specific antibodies, primary antibodies were diluted in Tris-buffered saline (TBS), pH 7.5, containing 5% (w/v) IgG-free BSA and 0.1% (v/v) Tween-20, and all washes were with TBS/0.1% (v/v) Tween-20. Quantification of immunoblots was by densitometric scanning of non-saturating films using Totallab v2.0 imaging software (Phoretix, Newcastle upon Tyne, UK).

### siRNA-mediated knockdown

HUVECs were plated out at a density of 2 × 10^5^ cells/ml in six-well plates. The next day, cells were transfected with 15 nM non-targeting or human Mcl-1-targeted siRNA in a total volume of 0.8 ml EGM-2 per well using 12 *μ*l HiPerFect. After 3 h, 1.6 ml/well EGM-2 was added and the cells left for 48 h before analysis.

### Clinicopathological details of ER^+^ cohort

A total of 195 patients diagnosed with ER^+^ ductal breast adenocarcinoma were involved in the study. All patients were treated solely with tamoxifen (104, 53.3%) or tamoxifen plus radiotherapy (34, 17.4%) or tamoxifen plus chemotherapy (35, 17.9%) or tamoxifen plus radiotherapy plus chemotherapy (22, 11.3%) according to protocols at the time of diagnosis. Median age of the patients was 64 with interquartile range from 54 to 71. According to pathological grading, 40 (20.5%) cases were first grade, 98 (50.3%) cases were second grade and 57 (29.2%) cases were third grade tumours. Median tumour size was determined to be 23 mm with interquartile range from 16 to 33 mm. Analysis of lymph nodes indicated that 99 patients (50.8%) had metastasis in comparison with 81 patients (41.5%) that had no metastasis in the lymph nodes. According to last patient follow-up dates, 101 (51.8%) patients were alive, 62 (31.8%) patients died of breast cancer and 32 (16.4%) patients died of another cause.

### TMA construction

In all, 0.6 mm^2^ cores of breast cancer tissue were removed from formalin-fixed paraffin-embedded tissue samples to construct the TMA blocks. All TMA blocks were constructed in triplicate containing three individual tumour cores taken from the same embedded tissue sample.

### Immunohistochemistry

Immunostaining with specific antibodies and validation of antibody specificity have been previously reported for this cohort of patients.^44, 45^

### *In situ* apoptosis detection assay

ApopTag Plus Peroxidase *In Situ* Apoptosis Detection Kit (Millipore, Billerica, MA, USA) was used to detect apoptotic cells. Sections were de-waxed in xylene and rehydrated in ethanol and water. Antigen retrieval was achieved by incubation for 15 min at 25 °C with 20 *μ*g/ml proteinase K (Millipore) in PBS, pH 7.4. Endogenous peroxidase activity was inactivated by incubating sections in 3% (v/v) hydrogen peroxide for 5 min at room temperature. Positive controls were generated by incubation for 30 min at 37 °C with 0.1 U/*μ*l RQ1 RNase-free DNase (Promega, Madison, WI, USA). To terminate the reaction, sections were incubated for 10 min at 65 °C in 1 : 50 stop solution (Promega) diluted in PBS. Further steps were followed as described by the supplier. Signals were visualised using 3,3'-diaminobenzidine (Vector Laboratories, Burlingame, CA, USA). Sections were then counterstained in Mayer's haematoxylin and dehydrated in ethanol and xylene before mounting. Apoptotic indices were calculated by dividing the number of apoptotic cells by the number of non-apoptotic cells and multiplying by 100.

### Scoring and statistical analyses

For the IHC experiments, protein expression was assessed using the weighted histoscore method described by Tovey *et al.*^56^ Protein expression was evaluated by two independent observers and the agreement between observers was assessed utilising interclass correlation analysis. Statistical analyses were performed using SPSS 19.0 (Chicago, IL, USA). Mann–Whitney *U*-tests were utilised to compare the expression of markers between different subgroups. Data from CCL39 cells and HUVECs are presented as means±standard error for the number of experiments indicated, while representative immunoblotting and FACS experiments are shown in the figures. Statistical significance was assessed either by one-way ANOVA or unpaired *t*-tests with an *α* probability of 0.05. At least three separate experiments were used for analysis.

## Abbreviations

S1P: D-*erythro*-sphingosine-1-phosphate
SK: sphingosine kinase
MS: multiple sclerosis
ERK: extracellular signal-regulated kinase
PI3K: phosphatidylinositol-3-kinase
PKC: protein kinase C
BH3: Bcl-2 homology domain 3
Bak: Bcl-2 homologous antagonist/killer
Bax: Bcl-2-associated X Protein
Bcl-2: B-cell lymphoma protein 2
Bcl-X_L_: Bcl-2-like protein 1
Bad: Bcl-X_L_/Bcl-2-associated death promoter
Bim: Bcl-2-interacting mediator of cell death
Mcl-1: myeloid cell leukaemia sequence 1
PUMA: p53-upregulated modulator of apoptosis
Bmf: Bcl-2-modifying factor
EC: endothelial cell
PI: propidium iodide
HUVEC: human umbilical vein endothelial cell
TMA: tissue microarray
ER: oestrogen receptor
DHS: D,L-*threo*-dihydrosphingosine
MEK: mitogen-activated protein/ERK kinase
PKB: protein kinase B
GSK3: glycogen synthase kinase 3
